# Impact of war on child health in northern Syria: the experience of Médecins Sans Frontières

**DOI:** 10.1007/s00431-017-3057-y

**Published:** 2017-12-19

**Authors:** Lana Meiqari, Maartje Hoetjes, Louisa Baxter, Annick Lenglet

**Affiliations:** 1 Médecins Sans Frontières, Operational Centre Amsterdam (MSF-OCA), Plantage Middenlaan 14, 1018 DD Amsterdam, The Netherlands; 2 Athena Institute for Research on Innovation and Communication in Health and Life Sciences, Vrije Universiteit Amsterdam, Amsterdam, The Netherlands; 3 Public Health England (PHE), London, UK

**Keywords:** Armed conflicts, Humanitarian assistance, Child health, Public health surveillance, Syria

## Abstract

Few data are available to evaluate the impact of Syrian war on civilian population; to describe this impact on child health, this article uses data from Médecins Sans Frontières-Operational Centre Amsterdam’s activities in Tal-Abyad and Kobane cities, northern Syria (2013–2016). Data were obtained from routine medical datasets and narrative reports, for out-patient clinics, immunisation, nutritional monitoring and assessments, and in-patient care, and were analysed quantitatively and qualitatively. Infections were the largest contributor to morbidity. The proportion of < 5 year out-patient consultations of infectious diseases that are listed for outbreak monitoring in emergencies was 15% in 2013, 51% in 2014, 75% in 2015 and 70% in 2016. Thalassemia was recorded in 0.5% of 2014 < 5 year out-patient consultations and 3.4% of 2013–2014 < 18-year in-patient admissions. Measles immunisation activities and routine Extended Programme for Immunisation were re-activated across northern Syria; however, immunisation coverage could not be calculated. Results from our routine data must be compared cautiously, due to differences in settings and disease categories.

*Conclusion*: With such scattered interventions, routine data are limited in providing a quantified evidence of emergency’s health impact; however, they help in drawing a picture of children’s health status and highlighting difficulties in providing curative and preventive services, in order to reflect part of population’s plight.

**Table:** 

**What is Known** *• Few data exist to evaluate the impact of the Syrian war on the health of children;* *• Médecins Sans Frontières (MSF-OCA) has worked in northern Syria during different times since 2013.*
**What is New** *• Quantitative and qualitative analysis of MSF’s routine medical data and situtation reports show that one fifth of all consultations in children < 5 years in MSF health facilities in northern Syria 2013–2016 were due to communicable diseases;* *• The analysis also highlights the burden of chronic conditions that were prevalent in Syria before the war,* e.g. *thalassemia.*

## Introduction

Children are extremely vulnerable during forced displacement and humanitarian emergencies. According to UNICEF (United Nations Children’s Fund), 28 million children are forcibly displaced worldwide due to violence and conflict. Nearly 65% of these children are displaced internally within their own countries, with the reminder externally displaced as refugees or asylum seekers [25]. The Syrian conflict has increased these numbers to an unprecedented level. To date, an estimated 8.5 million Syrian children have been forcibly displaced, with 71% displaced internally [23, 27].

Few data are available to evaluate the impact of this crisis on the health of Syrian children. An analysis of violent deaths among Syrian civilians has shown that women and children accounted for 25% of the number of civilians killed by weapons between March 2011 and April 2014 [8]. In addition to the direct effects of violence, the humanitarian needs in Syria and in neighbouring countries hosting refugees have been increasing due to repeated displacements of populations, severely damaged health systems, the displacement of medical professionals and restricted access for humanitarian actors [1, 2, 6, 7, 17]. The humanitarian needs for shelter, water and sanitation, food and healthcare among Syrian populations are expected to keep rising as the current crisis continues [3].

Médecins Sans Frontières-Operational Centre Amsterdam (MSF-OCA) has provided primary and secondary healthcare for populations in northern Syria during specific periods since 2013. We have analysed available medical and humanitarian data from these time periods to describe the impact of the crisis on children who attended our health facilities.

## Methods and materials

### Study location

**Fig. 1:**
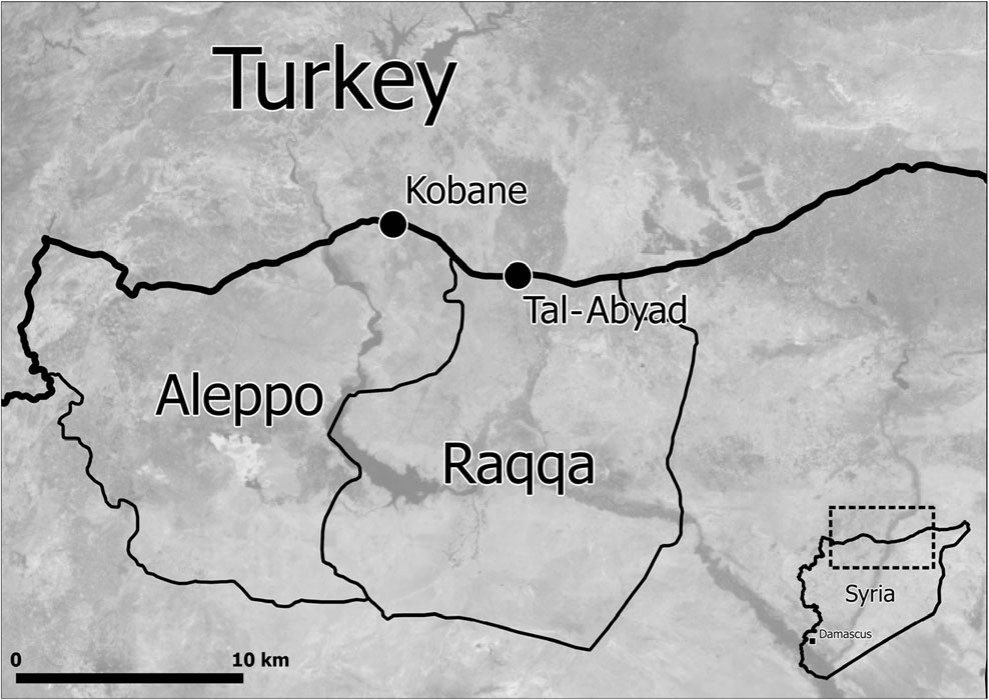
Location of Tal-Abyad and Kobane cities in Northern Syria, where Médecins Sans Frontières-Operational Centre Amsterdam (MSF-OCA) has been working since 2013

The districts of Tal-Abyad in Ar-Raqqa Governorate (estimated population 127,270 in 2004) and Kobane in Aleppo Governorate (Ayn El-Arab; estimated population 793,514 in 2004) are located in northern Syria close to the border with Turkey (Fig. 1) [21]. This region’s population comprises a mix of Arabs, Kurds and Armenians. During recent years, both districts have been taken over by different armed groups, leading to heavy fighting, sieges in the main cities and massive population displacements. Very few humanitarian organisations have retained a significant presence in both districts.

Since 2013, MSF-OCA has provided medical activities in these two districts, both within Tal-Abyad and Kobane cities and in surrounding villages via established clinical facilities and mobile teams. These activities have included out-patient clinics (OPD), in-patient care (IPD), vaccination activities through the Extended Programme of Immunisation (EPI) and supplementary immunisation activities (SIA) and nutritional monitoring and assessments.

### Quantitative analysis

#### Data sources

Routinely collected medical data were extracted from the MSF-OCA’s Health Information System. OPD data comprised weekly tally sheets recording the number of consultations by morbidity and by age (i.e. < 5 and ≥ 5 years). The morbidities include 25 fixed conditions with established case definitions (Table 1), and field medical staff can add ten optional conditions as needed. This article analyses OPD data for children < 5 years only.

**Table 1:** List of predefined primary diagnosis codes/conditions of the Health Information System (HIS) of Médecins Sans Frontières-Operational Centre Amsterdam (MSF-OCA), used at out-patient clinics (OPD) and in-patient care (IPD) classified by the main reason for their inclusion

Out-patient clinics (OPD)	In-patient care (IPD)
Outbreak/epidemic potential	
1. Severe diarrhoea/cholera 2. Bloody diarrhoea 3. Suspected/confirmed meningitis 4. Suspected/confirmed measles 5. Acute jaundice	1. Severe diarrhoea/cholera 2. Bloody diarrhoea 3. Suspected/confirmed meningitis 4. Suspected/confirmed measles 5. Acute jaundice
Preventative intervention monitoring	
6. Suspected neonatal tetanus 7. Acute flaccid paralysis	6. Suspected neonatal tetanus 7. Acute flaccid paralysis
High impact on morbidity/mortality	
8. ALRTI 9. Confirmed malaria 10. Acute watery diarrhoea	8. ALRTI 9. Confirmed malaria 10. Tuberculosis 11. AIDS-related illness
Population monitoring	
11. Suspected typhoid 12. Severe acute malnutrition 13. Chronic disease 14. Violence-related injury 15. Sexually transmitted infections 16. Gynaecological	12. Suspected typhoid 13. Severe malnutrition 14. Chronic disease 15. Violence-related injuries 16. Neonatal disease
Common complaint	
17. Anaemia 18. AURTI 19. Urinary tract infection 20. Eye infections	17. Anaemia 18. Fever of unknown origin
Public health surveillance	
21. Fever without identified cause 22. Suspected tuberculosis	
Further categories	
23. Common psychiatric disorders 24. Severe psychiatric disorders 25. Other	19. Other

*ALRTI* acute lower respiratory tract infection, *AURTI* acute upper respiratory tract infection

IPD data include the following information for each admission: patient identifier, date of admission, location referred from, age (for infants, converted to year ratios—e.g. 1 month = 0.08 years, 6 months = 0.50 years), sex, primary diagnosis code, details of primary diagnosis (as a free text), date of exit, exit code (four categories: discharged, defaulter, referred to other structures and death) and time between admission and death (three categories: < 24 h, ≥ 24 to < 48 h and ≥ 48 h). The primary diagnosis codes for IPD data include 19 conditions with established case definitions (Table 1). Ten optional codes can be added to these based on field medical staff needs. This article analyses IPD data for patients < 18 years only. Nutritional data were extracted from OPD records. Each OPD performs screening for mid-upper arm circumference (MUAC) for all children aged 6–59 months attending the clinic. Available data in this analysis reflect the number of children screened and the proportion of those with malnutrition. Severe acute malnutrition is defined as children with a MUAC < 115 mm and/or bilateral foot oedema. General acute malnutrition is defined as MUAC < 125 mm.

Information on vaccination was extracted from weekly tally sheets used to monitor EPI activities conducted within MSF-OCA facilities at Tal-Abyad (three sites in 2014, and five sites in 2016), and Kobane (five sites in 2015–2016). Data collected include the total number of doses of each vaccine administered. Reported vaccines include the following: Bacillus Calmette–Guérin for all ages, hepatitis B at birth (dose: 0), diphtheria, pertussis, and tetanus plus hepatitis B plus haemophilus influenza type B (doses: 1, 2, 3, booster), oral polio (doses: 0, 1, 2, 3, booster), intramuscular polio, measles (doses: 1 for 6–11 months, and 2 for ≥ 12 months) and measles/mumps/rubella. Individual-level immunisation data were not available to permit estimation of immunisation coverage in the target population.

#### Data management and analysis

In OPD data, we defined epidemic-prone diseases based on nine fixed categories (i.e. severe diarrhoea/cholera and acute watery diarrhoea, bloody diarrhoea, suspected/confirmed meningitis, suspected/confirmed measles, suspected neonatal tetanus, acute flaccid paralysis, upper and lower respiratory tract infections and suspected typhoid), and one optional category (i.e. acute jaundice syndrome). Other optional conditions, added by medical staff to OPD and/or IPD tally sheets, include the following: five chronic diseases (i.e. epilepsy, asthma, musculoskeletal pain, diabetes and hypertension), skin infections and non-violent injury. The optional morbidity categories differed across facilities and timeframes; thus, we re-coded them for the purposes of this analysis. Here are examples for recoded categories. Gastritis was originally coded in a separate category, but then coded within gastro-oesophageal reflux disease. Gastro-oesophageal reflux disease was recorded as a separate category in 2013 and 2014 only. Cutaneous leishmaniasis was not recorded in 2013. Acute watery diarrhoea and severe diarrhoea/cholera were merged into one category as watery diarrhoea. Acute respiratory tract infection included both upper and lower infections. Common and severe psychiatric disorders were merged. Sex was only available within OPD data for 2015–2016. Thalassemia was recorded at OPDs in 2014 and at Tal-Abyad’s paediatric IPD in 2013–2014. For IPD data, the free-text describing the primary diagnosis code of “other” was reviewed for all patients and this information was used in re-coding the primary diagnosis code when possible.

Data analysis focused on calculating proportional morbidities for patients < 5 years from OPD data and patients < 18 years for IPD data. Using the MUAC data, the prevalence of malnutrition was calculated by facility. Given the population’s dynamic displacement and movement, it was impossible to estimate a denominator for the target population. Therefore, the investigation of incidence, prevalence, coverage and healthcare utilisation was beyond the remit of our analysis. Quantitative data were cleaned and analysed using R software and Microsoft Excel.

### Qualitative data

#### Data sources

Data were extracted from available narrative reports which included situation reports (i.e. contextual and programmatic updates) and weekly or monthly medical reports. MSF-OCA field teams prepared these reports between April 2013 and September 2016.

#### Data management and analysis

Narrative sources were coded using MaxQDA software for information on: context, general observations at facilities, OPD and IPD consultations, immunisation activities (i.e. EPI and SIAs), surveillance and outbreak responses (with focus on polio and measles), nutrition, referrals and child injuries.

## Results

### Contextual description

**Fig. 2:**
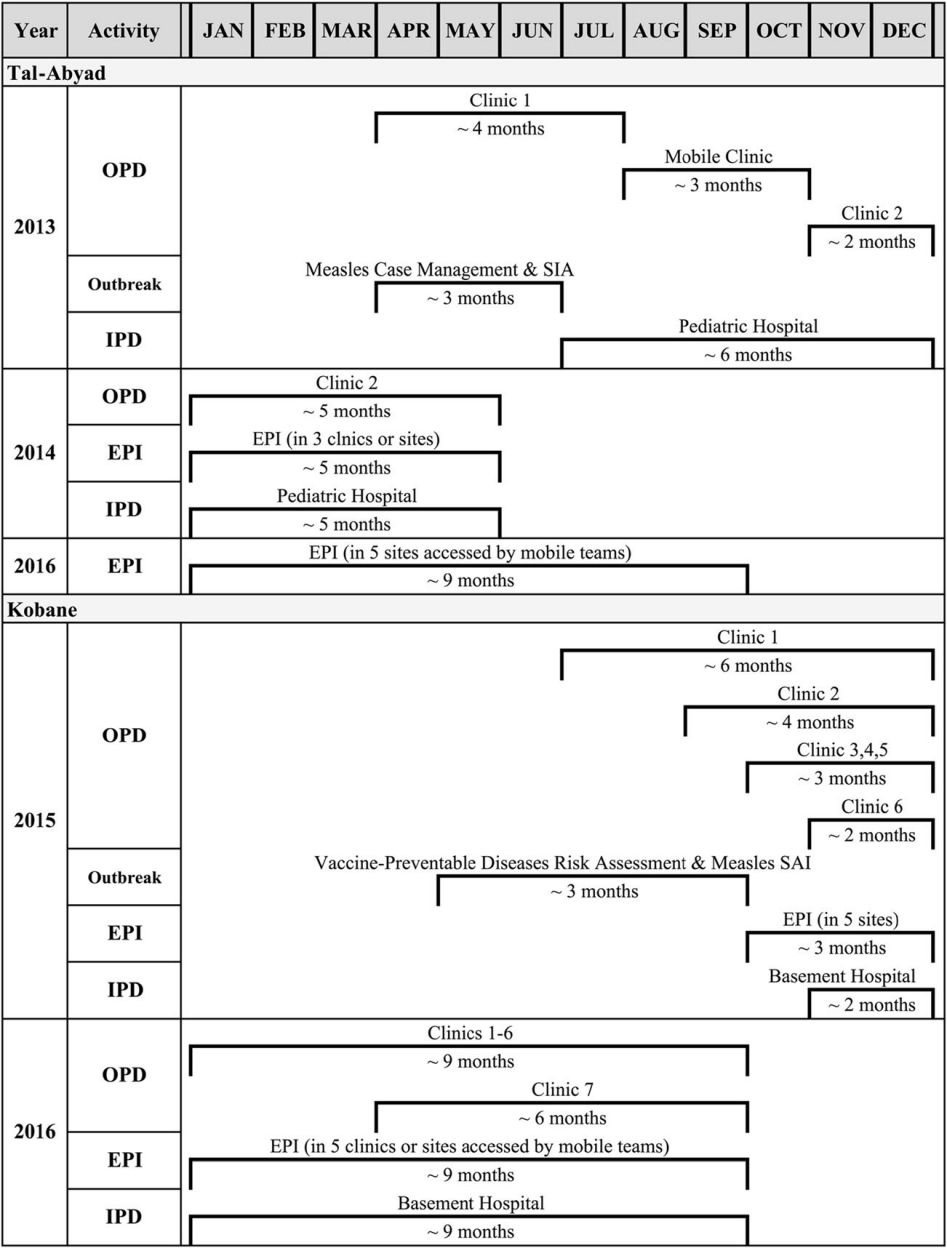
Timeline of Médecins Sans Frontières-Operational Centre Amsterdam (MSF-OCA) medical activities in Northern Syria, 2013–2016. SIAs supplementary immunisation activities. EPI expanded program on immunisation

A timeline of the medical activities at MSF-OCA in Tal-Abyad and Kobane between 2013 and 2016 is shown in Fig. 2. As the frontlines shifted in northern Syria during this period, MSF-OCA health facilities closed or opened suddenly, depending on security constraints and access. In April 2013, MSF-OCA intervened in Tal-Abyad by opening an OPD, then in July operating a paediatric IPD within Tal-Abyad National Hospital. Due to fighting, this OPD was then destroyed in August 2013 and subsequently converted into a mobile clinic until a new OPD was opened in October 2013 for 8 months. However, we could only retrieve data pertaining to 6 months of the clinic’s operation for this analysis. During this time, vaccination teams supported the re-activation of EPI from different centres across Tal-Abyad and AR-RAQQA districts. Following the evacuation of international staff in January 2014, the OPD and paediatric IPD and EPI activities were provided by Syrian healthcare staff until all activities ceased in May 2014.

Shortly after MSF-OCA re-entered northern Syria through Kobane in March 2015, the MSF-OCA team observed that over 15 villages had access to one healthcare facility, provided by a private clinic. The private doctor at this clinic reported treating an average of 15 patients per day at a reduced fee, while having no access to electricity and paying high costs for trucked water. Once a week, he allowed a Red Crescent team, of one doctor and four nurses, to treat patients for free (approximately 300 per week). The clinic reported having limited medications and medical supplies.

One month later, MSF-OCA established its first OPD in Kobane and continued to open new clinics in the city and surrounding villages, with a total of seven OPDs by 2016. In addition, MSF-OCA started IPD activities in November 2015 at a newly set up location referred to as Basement Hospital. Following a vaccine-preventable diseases risk assessment [5], EPI activities were initiated at MSF-OCA facilities and through mobile teams to access sites in both Kobane and Tal-Abyad districts.

### Morbidities at OPD and IPD

**Fig. 3:**
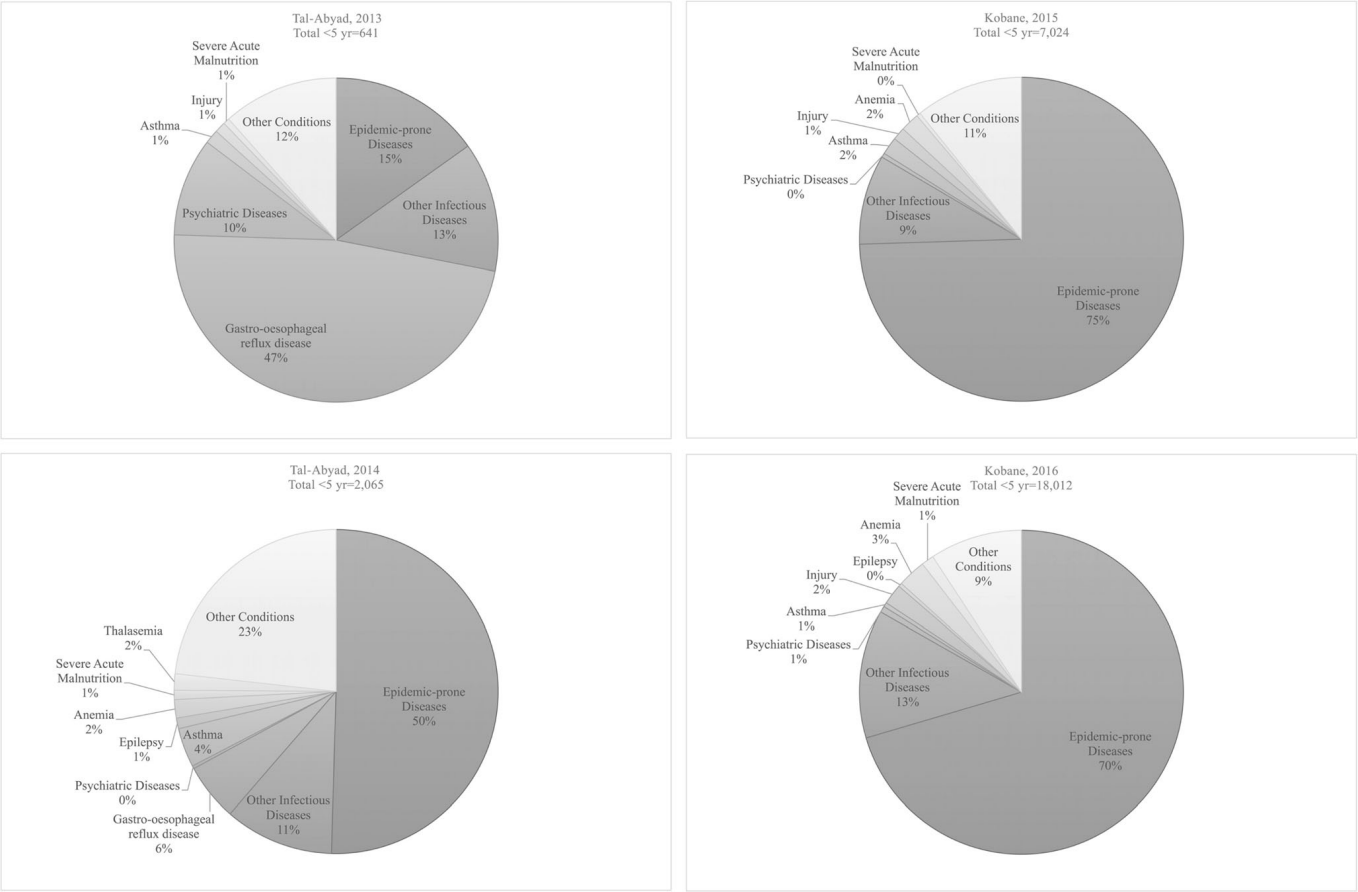
Morbidity trends of among consultations of children < 5 years old at out-patient facilities (OPD) for Médecins Sans Frontières-Operational Centre Amsterdam (MSF-OCA) in Northern Syria, 2013–2016. Epidemic-prone diseases include: severe diarrhoea/cholera and acute watery diarrhoea, bloody diarrhoea, suspected/confirmed meningitis, suspected/confirmed measles, suspected neonatal tetanus, acute flaccid paralysis, upper and lower respiratory tract infections, and suspected typhoid, and acute jaundice syndrome. Other infectious diseases category includes suspected tuberculosis, unidentified fever, urinary tract infection, skin infection and cutaneous leishmaniasis. Injury category includes injury and burns

Children < 5 years old constituted 15% (*n* = 641/4211) of total OPD consultations in 2013, 30% (*n* = 2065/6824) in 2014, 28% (*n* = 7024/24,921) in 2015 and 26% (*n* = 18,012/68,277) in 2016. Figure 3 shows an overview of the registered morbidities during the analysed period. Among children < 5 years in northern Syria, the majority of consultations were attributed to infectious diseases, such as acute respiratory tract infections (especially between November and February) and watery diarrhoea (especially between June and August). The proportion of consultations from epidemic-prone diseases in children increased from 15% (*n* = 97/641) in 2013 to 51% (*n* = 1042/2065) in 2014, 75% (*n* = 5233/7024) in 2015 and 70% (*n* = 12,684/18,012) in 2016. Other diseases constituting a high proportion of the overall morbidity data between 2013 and 2014 included gastro-reflux disease, asthma and skin infections.

In Tal-Abyad’s paediatric IPD (2013–2014), the number of admissions for patients < 18 years was 1823, and the mortality rate as a crude proportion of the total admissions was 1% (Table 2). Of overall admissions, 82% were in children < 5 years. In Kobane Basement IPD (2015–2016), the number of admissions for patients < 18 years was 2849 (59% of total admissions), and the mortality rate was 0.7%. Of overall admissions, 73% were in children < 5 years (Table 2). Gastrointestinal infections and lower respiratory tract infections were among the top five morbidities for IPD admissions across all age groups. In both hospitals, the highest mortality rate was observed in children < 6 months (1.3 and 2.8%). Malnutrition, diarrhoea and lower respiratory tract infections were the top three causes of death among children < 1 year.

**Table 2:** Admissions of those <18 years old at in-patient facilities (IPD) for Médecins Sans Frontières-Operational Centre Amsterdam (MSF-OCA) in Northern Syria, 2013–2016

	Tal-Abyad, Paediatric Hospital (Jul 2013–May 2014); total admissions = 1823						Kobane, Basement Hospital (Nov 2015–Sep 2016); total admissions = 2849					
	< 18 years	< 6 months	6 months–< 1 year	1–< 5 years	5–9 years	10–17 years	< 18 years	< 6 months	6 months–< 1 year	1–< 5 years	5–9 years	10–17 years
Admissions, *n* (%)												
	1823 (100%)	290 (16%)	445 (24%)	755 (41%)	210 (12%)	123 (7%)	1688 (59%)	365 (13%)	319 (11%)	551 (19%)	222 (8%)	231 (8%)
Sex, *n* (%)												
Male	1101 (60%)	180 (62%)	278 (63%)	433 (57%)	130 (62%)	80 (65%)	949 (56%)	204 (56%)	168 (53%)	316 (57%)	131 (59%)	130 (56%)
Top five morbidities (%)												
	Gastro (31%)	LRTI (42%)	Gastro (46%)	Gastro (29%)	Gastro (23%)	Gastro (23%)	Gastro (37%)	Gastro (29%)	Gastro (52%)	Gastro (42%)	Gastro (32%)	Gastro (25%)
	LRTI (26%)	Gastro (20%)	LRTI (32%)	LRTI (24%)	Thalassemia (11%)	Thalassemia (17%)	LRTI (18%)	LRTI (28%)	LRTI (22%)	LRTI (16%)	Other (15%)	Other (22%)
	URTI (5%)	Neonatal (13%)	Malnutrition (5%)	Seizure (7%)	LRTI (11%)	LRTI (8%)	Other (14%)	Other (14%)	Other (10%)	Other (14%)	LRTI (13%)	V. injury (12%)
	Seizure (5%)	Malnutrition (8%)	Fever (4%)	URTI (6%)	Other (9%)	URTI (8%)	Fever (9%)	Neonatal (11%)	Fever (8%)	Fever (9%)	Fever (11%)	Fever (10%)
	Other (4%)	Sepsis (4%)	Other (3%)	Intoxication (6%)	Asthma (8%)	Diabetes (8%)	Neonatal (3%)	Fever (6%)	Neonatal (3%)	Intoxication (3%)	V. injury (5%)	LRTI (6%)
Days admitted, *n*												
Mean	2.4	2.8	2.7	2.2	2.0	1.7	1.2	1.3	1.2	1.1	0.9	1.2
Median	2	2.0	2.0	2.0	1.0	1.0	1.0	1.0	1.0	1.0	1.0	1.0
Max	35	22	35	33	28	12	12	12	12	12	6	7
Exits status, %												
Discharges	83.8	78.6	86.7	84.6	85.7	76.4	86.3	83.8	90.3	85.8	86.0	85.7
Defaulter	9.7	8.3	8.3	10.9	7.6	14.6	9.7	8.2	8.5	12.0	10.8	7.4
Referred	4.6	9.3	2.9	3.4	4.3	6.5	3.1	5.5	0.3	2.0	2.3	6.5
Death	1.0	2.8	0.9	0.4	0.5	2.4	0.7	2.2	0.9	0.0	0.5	0.0
Missing	0.9	1.0	1.1	0.7	1.9	0.0	0.2	0.3	0.0	0.2	0.5	0.4
Diagnosis for death cases												
		Malnutrition (*n* = 4)	Jaundice	Anaemia	Coma	Thalassemia		Malnutrition	LRTI (*n* = 2)		Heart disease	
		Diarrhoea (*n* = 2)	Diarrhoea	Heart disease		Intoxication		Jaundice (*n* = 2)	Diarrhoea			
		Neonatal	Fever	Intoxication				LRTI				
		Meningitis	LRTI					Seizure				
		Other^a^						Diarrhoea				
								Sepsis				
								Neonatal				

^a^Other is only detailed as “persistent vomiting after surgery”

*Gastro* gastro-enteritis, *LRTI* lower respiratory tract infection, *URTI* upper respiratory tract infection, *Neonatal* neonatal diseases, *V. Injury* violent injury

Thalassemia cases accounted for 0.5% of 2014 OPD consultations for children < 5 years, and 3.4% of Tal-Abyad paediatric IPD admissions 2013–2014; it was a common morbidity for children 5–9 and 10–17 years (11 and 17% of admissions, respectively).

There were only few records of violent injuries among children < 5 years within OPD and IPD data. However, situation reports from both Tal-Abyad and Kobane regularly mentioned cases of injuries due to unexploded ordnance and improvised explosive devices.

### Outbreak monitoring and immunisation

**Fig. 4:**
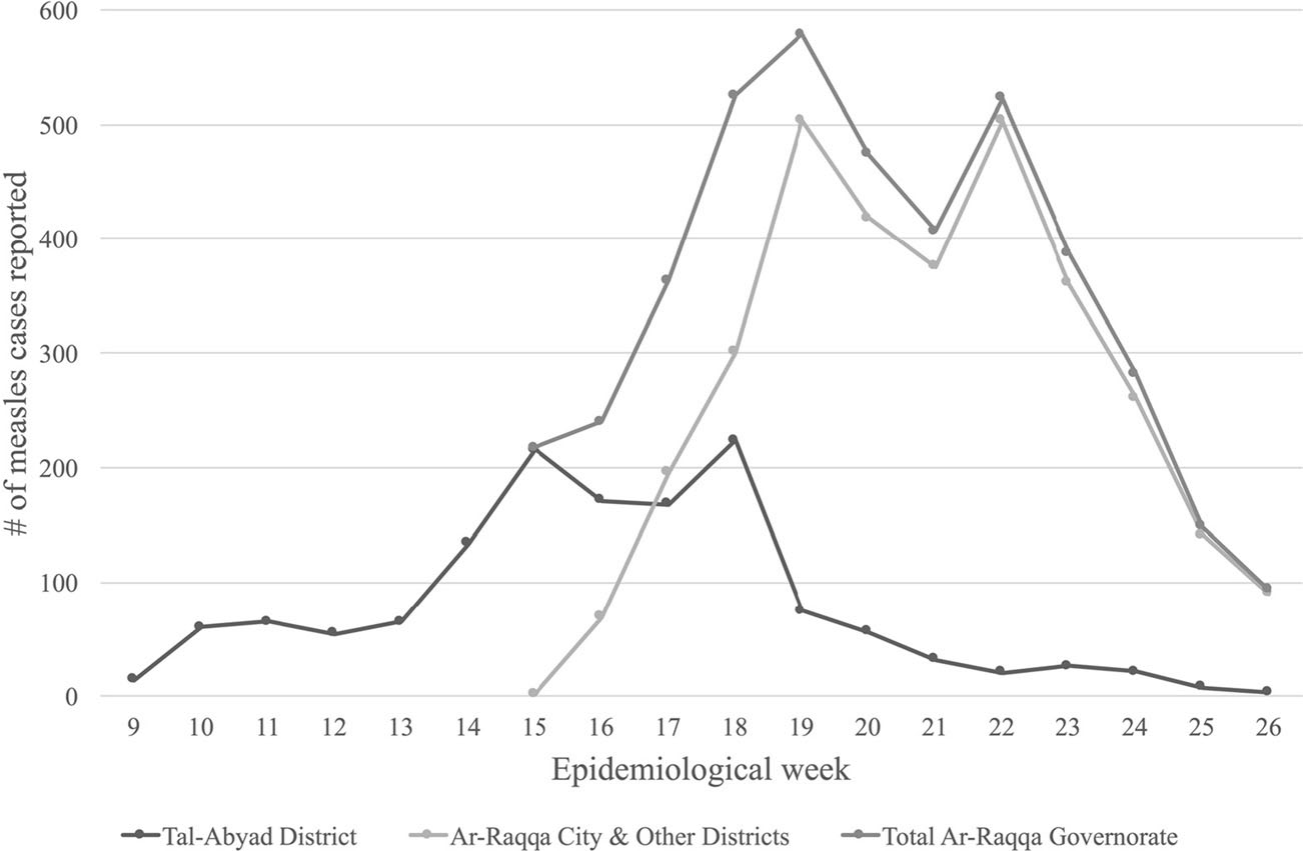
Epidemiological curve for measles cases reported in Tal-Abyad and other districts of Ar-Raqqa governorate during an outbreak response by Médecins Sans Frontières-Operational Centre Amsterdam (MSF-OCA) in Northern Syria, 2013

In 2013, MSF-OCA responded to a measles outbreak in Tal-Abyad, with mobile teams for measles treatment and outbreak monitoring. The teams covered Tal-Abyad district and other districts of Ar-Raqqa (Fig. 4). The total number of reported measles cases in Ar-Raqqa governorate was around 4600. The majority of measles cases (61%) were < 5 years, with 28% of cases reported in children 5–14 years and 11% in > 15 years of age. The proportion of cases in the 5–14 years age group was higher in other districts of Ar-Raqqa compared to Tal-Abyad district (35 vs. 14%). In contrast, the proportion of cases in the < 5 years age group was higher in Tal-Abyad district compared to other districts of Ar-Raqqa (75 vs. 54%).

In response to this outbreak, MSF-OCA teams planned and conducted measles SIA only within Tal-Abyad district, due to the insecure situation in other districts. The campaign vaccinated 37,325 children 6–59 months during weeks 21 to 24 which was higher than the estimated target population of 35,335 children, resulting in a calculated vaccination coverage of 106%. Problems with population estimation were discussed in the medical report, week 26/27, June 2013:

The challenges have been the high security context, making some areas hard or impossible to reach; the difficulties of getting appropriate population data and working in a fluctuating population on the move; managing big crowds of people who are not used to cueing; and finding and reaching all the children in the scattered houses and tents.

To address these concerns, a catch-up campaign for hard-to-reach villages vaccinated 1135 children in epidemiological weeks 28 and 29. However, narrative reports in subsequent weeks continued to document the identification of non-vaccinated children, mainly in newly arrived families.

**Fig. 5:**
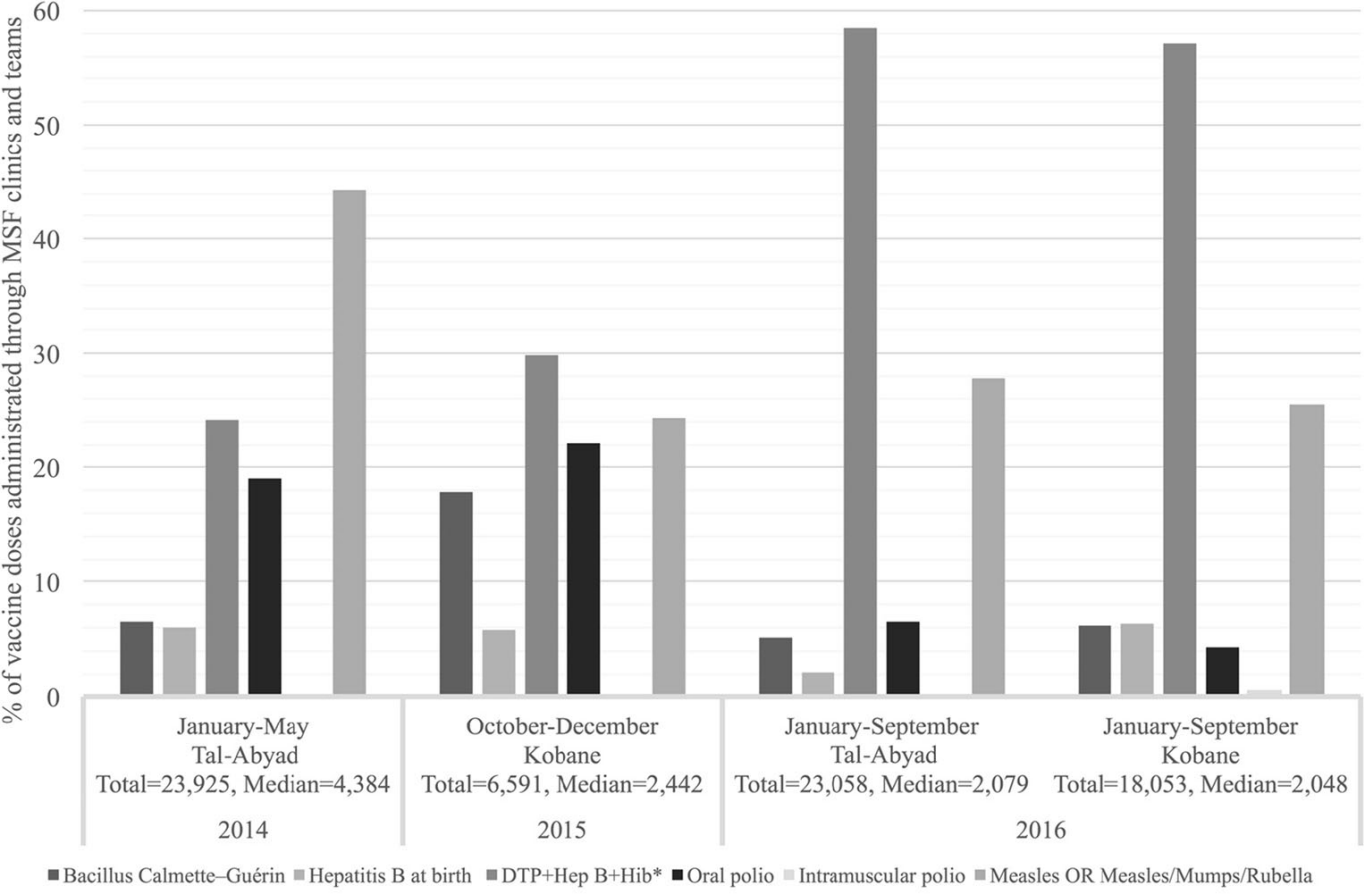
Immunisation activities of Médecins Sans Frontières-Operational Centre Amsterdam (MSF-OCA) facilities and mobile teams in Northern Syria, 2013–2016. Asterisk indicates diphtheria, pertussis (whooping cough) and tetanus (DTP) + Hep B + haemophilus influenza type B (Hib)

When MSF teams re-entered Syria in 2015, one of their first responses was to perform a vaccine-preventable diseases risk assessment and measles SIA in Kobane and surrounding villages [5]. Regarding EPI, Fig. 5 shows the proportion of vaccine doses by vaccine type administrated through MSF-OCA facilities and mobile teams.

MSF also implemented surveillance activities for cases of communicable diseases to detect possible outbreaks early. For example, in 2013, five cases of viral meningitis were reported in epidemiological week 21; cases of hepatitis A were reported between week 22 and week 25; and cases of leishmaniasis, brucellosis and typhoid were reported in weeks 26–27. Preventive measures followed on these reports. For example, MSF-OCA surveillance teams prepared factsheets and trained local medical doctors on the diagnosis and treatment of children with hepatitis A after suspected cases were reported at school buildings used to house internally displaced persons. Additionally, MSF-OCA teams improved the drainage, sanitation and cleaning of buildings and distributed health education materials about personal hygiene and sanitation at schools and at MSF-OCA facilities.

### Nutritional monitoring and assessment

The proportion of general acute malnutrition as measured by MUAC screening at MSF-OCA facilities and during SIAs ranged between 0.3% (April–December 2015) and 0.8% (January–May 2014). The proportion of severe acute malnutrition identified through SIAs’ community-based screening was lower (0.1%) than through health facility-based screening (range: 0.8–2.2%).

A high proportion of hospital admissions in Tal-Abyad’s paediatric IPD for children < 1 year was for severe acute malnutrition (13%). In addition, recorded deaths due to malnutrition within IPD data were among children < 6 months: 4 deaths in Tal-Abyad, and 1 death in Kobane (Table 2).

To explore malnutrition cases among infants < 1 year, MSF teams inquired about breastfeeding practices in a household survey (Kobane, June 2015), which reported low rates of exclusive breastfeeding:

Of the 19 children <6mo old, 3 of the 19 were exclusively breastfed (16%). Of the 12 mothers who did not breastfeed their child <1yr of age, 10 reported initiating of breastfeeding before stopping, while 2 never initiated breastfeeding. Reported reasons for stopping or not initiating are: mother had not enough breast milk (n=10); mother was ill or baby was not physically able to feed from mother’s breast (n=4); mother stopped after a traumatic experience (n=1); mother stopped because family told her to stop and start using powder milk (n=1).

## Discussion

This analysis of medical data from MSF-OCA’s health facilities in northern Syria for 2013–2016 shows that the main burden of disease and mortality in children < 5 years relates to infectious diseases and malnutrition. More than half of consultations for children < 5 years in OPD were for epidemic-prone diseases. This information is supported by surveillance systems established for the Syrian emergency, such as the EWARS/ EWARN systems [13, 15, 19], which reported outbreaks of vaccine-preventable diseases like measles and polio during the same time period [11, 14, 18].

Interruptions of routine EPI across northern Syria increased the risk of outbreaks and left a large proportion of children incompletely vaccinated, thus susceptible to infection. Estimates show that over 50% of children born during the Syrian conflict are unvaccinated [17] and that measles immunisation coverage for Syrian children aged 12–23 months dropped from 80% in 2011 to 54% in 2014 [12]. Strategies to increase the immunisation coverage will have a significant impact on reducing morbidity and mortality.

There is a high carrier rate of haemoglobinopathies among the Syrian population, including beta-thalassemia (5%) and alpha-thalassemia (1–5%) [9]. This rate is possibly higher among the Ar-Raqqa population due to consanguineous marriages [16], which may explain finding admissions and consultations for thalassemia patients at MSF-OCA’s facilities. However, the trends in relation to these admissions to IPD fluctuated over the months analysed. These fluctuations may reflect population mobility, whereby patients search for specialised care in the context of non-functioning or overwhelmed health facilities affected by low resources, overcrowding and security challenges [28, 29].

Undernutrition, anaemia and low breastfeeding rates were already known to be problematic in pre-war Syria [26]. Nutritional surveys conducted among Syrian refugees living in neighbouring countries in 2013–2014 showed a low level of general acute malnutrition (< 5%) among children 6–59 months [4, 10]. In northern Syria, our analysis found a higher proportion of severe acute malnutrition among children attending facilities, as compared to community samples, which suggests that those searching for healthcare were more likely to be malnourished. The high morbidity and mortality of malnutrition at IPD, especially among children < 1 year, suggests a link between malnutrition and inappropriate or inadequate infant-feeding practices in northern Syria; this might be a result of limited exclusive breastfeeding practices and difficulty in accessing baby formula milks. It is not possible with this analysis to fully evaluate malnutrition risk or prevalence and changes over time. However, recent reports from besieged Eastern Ghouta in rural Damascus have recorded higher rates of malnutrition; in October 2016, > 1100 children were reported to have acute malnutrition, and 72% of children < 5 years and pregnant and lactating women were reported as needing urgent nutritional support [20, 22, 24].

Moreover, a high burden of anaemia and other micronutrient deficiencies has been reported among Syrian refugees living in neighbouring countries; for children 6–59 months, anaemia prevalence was as high as 48.4% in a Jordanian refugee camp, 13.9% in one region of Lebanon [4, 10]. This analysis could not investigate anaemia since anaemia cases at MSF-OCA data were based on clinical signs and symptoms only, without access to data from laboratory diagnostics.

This is a large dataset (2013–2016) from one of the major humanitarian organisations working in northern Syria. Although robust statistical analysis was not possible, these results can inform research and policies regarding the health situation of children in northern Syria; for example, what further research questions are important and what medical interventions to be prioritised. Nevertheless, this analysis has key limitations; notably, data were collected under dynamic and volatile conditions, which included sudden opening or closure of health facilities, security concerns and displacement of populations. This dynamic context over the 4 years produces differences in patient groups and health priorities, including the need for specific data, which could have contributed to information bias and skewed results. A further source of bias could relate to data entry, which was done in different locations and by different medical staff, with changes over time in the precise category coding used. In addition, we did not have a continuous timeframe for our data; thus, results may not be representative of the complete time period. Other restrictions on our ability to extrapolate conclusions included the following: the absence of population denominators, differences in disease categorisation among health facilities and limited data pertaining to key diseases such as anaemia. It would be important to include further analysis of MSF-OCA data on mental health problems and war-related injuries in the paediatric population, since both conditions are known to result in long-term adverse effects for child health, education and social integration.

In conclusion, we have provided an overview of the daily reality for MSF-OCA facilities across northern Syria and illustrated the impact of this conflict on its most vulnerable, the children. For healthcare workers and systems in Europe and other host countries who are taking care of displaced/refugee children, we hope this overview and description of the breakdown of curative and preventive health services in children’s country of origin will help them to better detect and address the health needs of these children.

## Abbreviations

EPI: Extended Programme for Immunisation
IPD: In-patient care/department
MSF: Médecins Sans Frontières
MSF-OCA: Médecins Sans Frontières-Operational Centre Amsterdam
MUAC: Mid-upper arm circumference
OPD: Out-patient clinics/department
SIAs: Supplementary immunisation activities
